# Estimating the burden of seasonal influenza in Spain from surveillance of mild and severe influenza disease, 2010‐2016

**DOI:** 10.1111/irv.12499

**Published:** 2017-12-15

**Authors:** Jesús Oliva, Concepción Delgado‐Sanz, Amparo Larrauri, the Spanish Influenza Surveillance System, Virtudes Gallardo, Jose María Navarro, Elisa Marco, Manuel Omeñaca, Ismael Huertas, María de Oña, Jaume Giménez, Lucas González, Carmen Pérez, Luis Viloria, Mónica Gozalo, Gonzalo Gutiérrez, Tomás Vega, Socorro Fernández, Nuria Torner, María de los Ángeles Marcos, Aurora López, Francisco González, Concepción Gimeno, Julián Mauro Ramos, Guadalupe Rodríguez, María Jesús Purriños, Juan García Costa, Sonia Pérez‐Castro, Luis García, Juan Carlos Galán, Rocío García, Antonio Moreno, Jesús Castilla, Mirian Fernández‐Alonso, Carmen Ezpeleta, Fernando González Carril, Gustavo Cilla, Carmen Quiñones, Miriam Blasco, Ana Rivas, José López, Francisco Pozo, Inmaculada Casas, Salvador de Mateo, Raúl Ortiz de Lejarazu

**Affiliations:** ^1^ National Centre of Epidemiology CIBER Epidemiología y Salud Pública (CIBERESP) Institute of Health Carlos III (ISCIII) Madrid Spain

**Keywords:** burden disease, influenza, primary care, secondary care, Spain, surveillance

## Abstract

**Background:**

Estimating the national burden of influenza disease is challenging. We aimed to estimate the disease burden of seasonal influenza in Spain, at the primary care and hospital level, over the 6 influenza seasons after 2009 pandemic.

**Methods:**

We used data from the Spanish Influenza Sentinel Surveillance System to estimate weekly influenza rates and the number of influenza‐like illness (ILI) and mild confirmed influenza cases (MCIC). From the surveillance of severe hospitalized confirmed influenza cases (SHCIC), we obtained hospitalization rates and total number of SHCIC, intensive care unit (ICU) admissions and deaths in influenza hospitalized patients. We estimated both mild and severe influenza cases, overall, and by age‐group (<5, 5‐14, 15‐64, and ≥65 years).

**Results:**

The highest cumulative rates of MCIC were observed in <15 years (1395‐3155 cases/100 000 population in 5‐14 years) and the lowest in ≥65 years (141‐608 cases/100 000 population). SHCIC rates revealed a characteristic U‐shaped distribution, with annual average hospitalization rates of 16.5 and 18.9 SHCIC/100, 000 p in 0‐4 years, and ≥65 years, respectively. We estimated an annual average of 866 868 cases of ILI attended in primary care (55% were MCIC), 3616 SHCIC, 1232 ICU admissions, and 437 deaths in SHCIC. The percentage of ICU admission among SHCIC was highest at 15‐64 years (42%), while the hospitalization fatality rate ranged from 1% in 0‐4 years to 18% in ≥65 years.

**Conclusions:**

The ongoing Spanish Influenza Surveillance System allowed obtaining crucial information regarding the impact of mild and severe influenza in Spain.

## 1 INTRODUCTION

Seasonal influenza is an acute respiratory infection caused by worldwide circulating influenza viruses. The spectrum of disease severity ranges from mild forms of disease to those with severe complications, including death. The main aim of influenza immunization programs is the reduction of hospitalization and death occurring mainly among high‐risk individuals. However, the burden of mild outpatient illness is also important because it accounts for a large proportion of absenteeism in both healthy adults and children.^1^

Current estimates indicate that, worldwide, the annual influenza epidemics affect 5%‐10% of the world's population, resulting in about 3‐5 million cases of severe illness, and about 250 000‐500 000 deaths.^1^ The European Center for Disease Prevention and Control (ECDC) estimates that nearly 40 000 people in the European Union die prematurely each year due to influenza‐related causes.^2^

To better assess and plan targeted public health interventions, national estimates of the burden of seasonal influenza are needed to compare between different influenza seasons and pandemics.

Estimating the national burden of influenza disease is however challenging. Recently, the WHO issued recommendations toward the use of influenza surveillance data to better estimate the burden of disease at the national level.^3^

The Spanish Influenza Sentinel Surveillance System (SISSS) is a well‐established and consolidated system that provides timely information on the activity of mild influenza disease in Spain at primary care level since 1996.^4^, ^5^ It is based on sentinel physicians reporting clinical influenza‐like illness (ILI) cases and integrates virological data collected in the same population,^6^ providing an opportunity to calculate the disease burden in primary care. Regarding influenza severity, the surveillance system for severe hospitalized confirmed influenza cases (SHCIC) was established in Spain after the influenza pandemic of 2009, including sentinel hospitals from all Spanish regions.

To date, estimates of influenza‐associated disease burden in Spain have been focused on specific age‐groups,^7^ regional geographical areas,^8^ or severe disease but have not considered mild influenza disease.^9^ Our aim was to utilize data obtained by the SISSS and SHCIC on the surveillance of mild and severe disease at the primary and secondary care level to estimate the age related burden of seasonal influenza disease in Spain, in the post‐2009 pandemic period.

## 2 METHODS

### 2.1 The Spanish Influenza Sentinel Surveillance System (SISSS)

The SISSS comprises 17 networks of sentinel physicians (general practitioners and pediatricians) in 17 of the 19 Spanish regions, as well as the network‐affiliated laboratories, including the National Influenza Reference Laboratory (National Centre for Microbiology, World Health Organization National Influenza Centre in Madrid). More than 800 sentinel physicians participated each season covering a population under surveillance of around 1 million of physician's catchment area, giving an overall coverage of 2.2% of the total population of the 17 Spanish regions. All the networks complied with a series of requirements as to the minimum population covered (>1%) and representativeness in terms of age, sex, and degree of urbanization.^4^, ^5^

Sentinel physicians reported ILI cases detected in their reference populations on a weekly basis, following a definition based on the EU‐ILI case definition.^10^ For influenza surveillance, they systematically swabbed (nasal or nasopharyngeal) the first 2 ILI patients each week and sent the swabs to the network‐affiliated laboratories for influenza virus detection. The information collected in the SISSS includes data on demographic, clinical and virological characteristics, seasonal vaccination status, chronic conditions, and pregnancy. Data are entered weekly by each regional sentinel network in a web‐based application (http://vgripe.isciii.es/gripe) and analyzed by the National Centre of Epidemiology to provide timely information on the evolving influenza activity in Spanish regions and at the national level.

We used data provided by the SISSS for the 2010‐2011 to 2015‐2016 influenza seasons. We calculated the cumulative weekly ILI rates by season, for all ages and by age‐group (0‐4, 5‐14, 15‐64, and >64 years). By extrapolation to the Spanish population we estimated the number of ILI cases by age and season and by applying the corresponding weekly positivity rate, we estimated the number of mild confirmed influenza cases (MCIC). Point estimates and 95% confidence intervals (95% CI) were calculated.

### 2.2 Surveillance of SHCIC

The number of hospitals participating in the surveillance of SHCIC ranged between 181 in the post‐pandemic season 2010‐2011 and 90 in the 2013‐2014 influenza season. The population under surveillance reached 60% of the total Spanish population in the 2010‐2011 season and was maintained around 45% from the 2011‐2012 season onwards. The catchment population in the surveillance of SHCIC was the reference population of each participating hospital in each season, who would be referred to those hospitals for any reason leading to hospitalization. As the Spanish secondary health system provides national public coverage to almost all the Spanish population, the catchment hospital population is completely generalizable nationwide.

The system is based on the notification of those laboratory confirmed influenza hospitalized cases in all wards who met the Spanish SHCIC case definition. This case definition includes several severity criteria: “Any case with clinical features compatible with influenza, requiring hospitalization for clinical severity: at least one of the following criteria: pneumonia, septic shock, acute respiratory distress syndrome, multiple organ dysfunction syndrome, or admission to ICU.” Although it was recommended to swab all influenza cases needing to be hospitalized, in the clinical practice, the extent of respiratory swabbing might depend of the hospital physician's discretion. Following swabbing, only those cases that were influenza confirmed and meet the case definition were reported. Data on clinical, epidemiological, and virological information were collected, as well as the patient outcome. In this way, one unique system provides information on all hospitalized patients with severe confirmed influenza infection, including the ICU‐admitted and deceased.

We used data obtained from the surveillance of SHCIC for the same study period and age‐groups as in primary care, to calculate the cumulative hospitalization rates as well as the cumulative rates of ICU admission for each season. We used as denominators the population under surveillance in the participating hospitals. These rates were applied to the total Spanish population to estimate the total number of SHCIC and ICU‐admitted patients (including the 95% CI) for the whole country, by season and age‐group. The number of deaths in SHCIC was also extrapolated to the Spanish population to estimate the number of deaths in influenza hospitalized patients in Spain.

We used as denominators the Spanish population of the first year of each season (total and by age‐group), obtained from the Spanish National Statistics Institute.^11^

All estimations were performed using version 14.1 of the statistical analysis software stata (Stata Corp., College Station, TX, USA).

## 3 RESULTS

### 3.1 Seasonality of influenza activity in Spain through the Influenza Surveillance Systems in primary and secondary care

**Figure 1:**
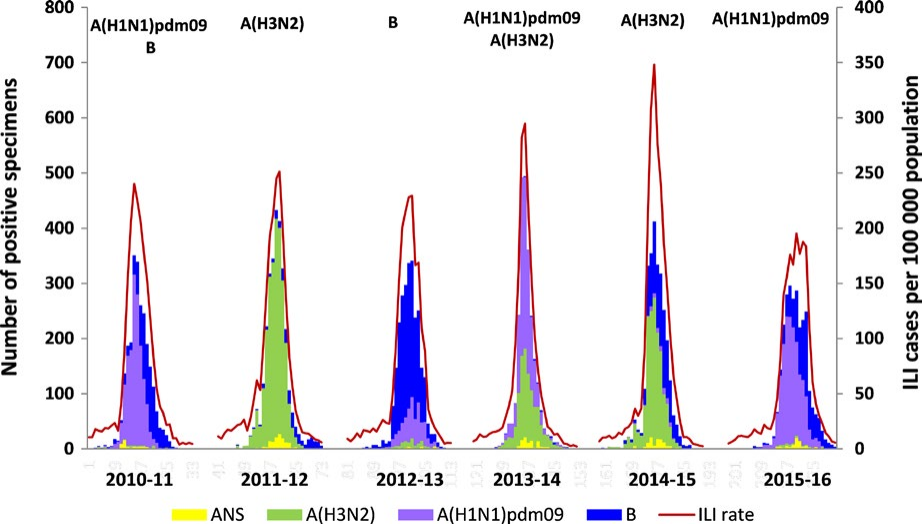
Primary care. Weekly ILI rates and number of type/subtype influenza virus by season, SISSS, Spain

Influenza activity in sentinel primary care varied by studied season (Figure 1). The ILI rates at the epidemic peak ranged between 348 ILI cases per 100 000 population, in the 2014‐2015 season, and 195 ILI cases per 100 000 population, in the 2015‐2016 season. There was a predominant circulation of A(H1N1)pdm09 in the 2010‐2011, 2013‐2014 (with co‐circulation of A[H3N2]), and 2015‐2016 season; influenza A(H3N2) predominated in the 2011‐2012, 2013‐2014 (co‐circulating with A[H1N1]pdm09), and 2014‐2015 season. The influenza B predominated in the 2012‐2013 season, although influenza B virus also circulated in a considerable extent following the peak of 2010‐2011, 2014‐2015, and 2015‐2016 influenza seasons. Three epidemics had a usual presentation on time reaching the peak in January (2010‐2011, 2013‐2014, 2014‐2015) and 3 peaked later, toward middle‐end of February (2011‐2012, 2012‐2013, and 2015‐2016).

**Figure 2:**
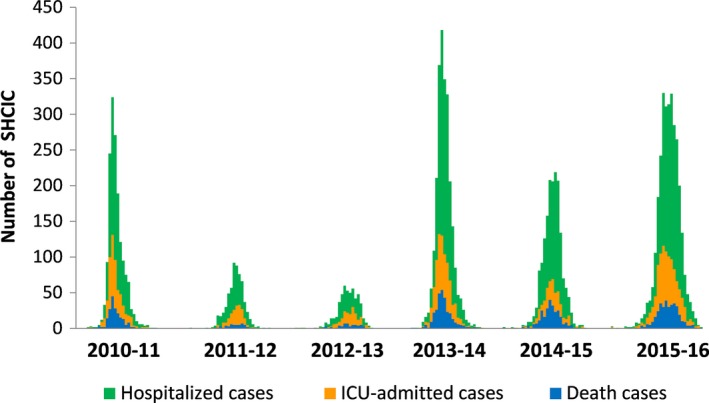
Secondary care. Weekly number of severe hospitalized confirmed influenza cases, ICU admissions and deaths in influenza hospitalized patients, surveillance of SHCIC, Spain

The weekly number of SHCIC, ICU admissions and deaths in influenza hospitalized patients along the 6 studied influenza seasons is shown in Figure 2. The total number of SHCIC ranged from 525 in the 2012‐2013 season to 3101 in the 2015‐2016 season.

We provide here results at national level. Influenza activity observed at primary and secondary health care in Spain varied by Spanish region and season and was timely reported through the national weekly and annual influenza reports.

### 3.2 Estimated influenza burden disease

#### 3.2.1 ILI cases and MCIC in sentinel primary care

**Figure 3:**
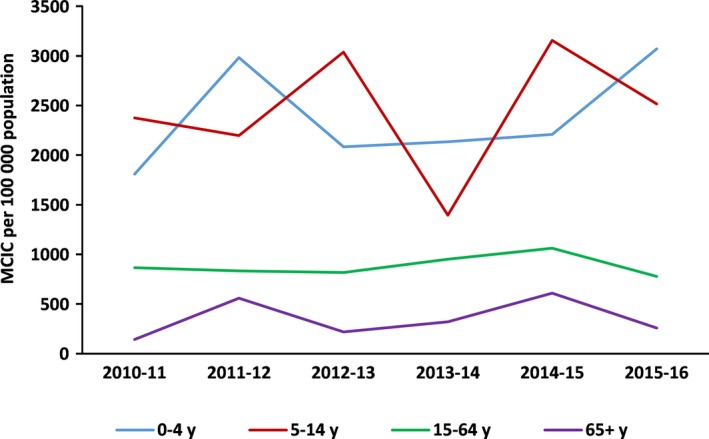
Primary care. Cumulative annual rates of mild confirmed influenza cases by age group, SISSS, Spain

The highest cumulative MCIC rates were observed in children under 15, whereas the lowest in the elderly (65+ aged) (Figure 3). The cumulative MCIC rates varied from 141 to 608 cases per 100 000 population in the elderly, depending on the season, and from 1395 to 3155 cases per 100 000 population in the 5‐ to 14‐year age‐group.

The number of ILI cases in Spain in the study period ranged between 792 783 in 2013‐2014 and 984 061 in 2014‐2015 influenza season; approximately 50% of them were MCIC ranging between 431 468 in 2010‐2011 and 580 331 in 2014‐2015 influenza season (Table 1).

**Table 1:** Influenza‐like illness (ILI) and mild confirmed influenza cases attended in primary care by age‐group. SISSS, Spain, seasons 2010‐2011 to 2015‐2016

Season	Dominant influenza type/subtype	ILI cases			Mild confirmed influenza cases		
		Number	95% CI		Number	95% CI	
All ages							
2010/11	A(H1N1)2009	860 490	858 673	862 310	431 468	430 182	432 757
2011/12	A(H3N2)	893 685	891 833	895 540	482 108	480 748	483 471
2012/13	B	844 907	843 106	846 711	464 483	463 148	465 821
2013/14	A(H1N1)2009	792 783	791 039	794 530	438 519	437 222	439 819
2014/15	A(H3N2)	984 061	982 118	986 007	580 331	578 839	581 826
2015/16	A(H1N1)2009	825 283	823 503	827 066	450 735	449 420	452 053
Aged 65+							
2010/11	A(H1N1)2009	41 054	40 658	41 453	11 096	10 890	11 304
2011/12	A(H3N2)	73 548	73 017	74 081	44 550	44 137	44 966
2012/13	B	45 865	45 446	46 287	17 708	17 448	17 971
2013/14	A(H1N1)2009	54 121	53 666	54 579	26 532	26 214	26 853
2014/15	A(H3N2)	88 673	88 090	89 259	51 228	50 785	51 674
2015/16	A(H1N1)2009	51 940	51 494	52 389	22 078	21 788	22 371
15‐64 y							
2010/11	A(H1N1)2009	532 282	530 853	533 714	270 056	269 038	271 076
2011/12	A(H3N2)	519 805	518 393	521 220	263 827	262 821	264 836
2012/13	B	473 313	471 966	474 663	256 924	255 931	257 919
2013/14	A(H1N1)2009	520 517	519 104	521 933	296 631	295 564	297 700
2014/15	A(H3N2)	578 879	577 389	580 372	329 361	328 237	330 488
2015/16	A(H1N1)2009	449 705	448 392	451 021	239 399	238 441	240 360
5‐14 y							
2010/11	A(H1N1)2009	192 861	192 001	193 724	106 189	105 551	106 830
2011/12	A(H3N2)	171 381	170 571	172 194	99 214	98 598	99 833
2012/13	B	219 807	218 889	220 728	139 215	138 485	139 948
2013/14	A(H1N1)2009	123 587	122 899	124 278	64 914	64 416	65 415
2014/15	A(H3N2)	219 132	218 215	220 051	148 999	148 243	149 758
2015/16	A(H1N1)2009	198 662	197 789	199 538	120 573	119 893	121 256
0‐4 y							
2010/11	A(H1N1)2009	94 292	93 691	94 896	44 127	43 716	44 541
2011/12	A(H3N2)	128 952	128 249	129 658	74 517	73 983	75 054
2012/13	B	105 922	105 285	106 562	50 636	50 196	51 079
2013/14	A(H1N1)2009	94 558	93 956	95 163	50 442	50 003	50 884
2014/15	A(H3N2)	97 377	96 766	97 991	50 744	50 303	51 187
2015/16	A(H1N1)2009	124 977	124 285	125 672	68 685	68 172	69 201

There were differences in the estimated burden of influenza in primary care by age. In elderly, we estimated the highest annual number of MCIC (from 44 550 to 51 228) in the A(H3N2) dominant influenza seasons 2011‐2012 and 2014‐2015, respectively (Table 1). The 5‐ to 14‐year age‐group was most affected in the B season 2012‐2013 (139 215) and also in the A(H3N2) season 2014‐2015 (148 999) and the A(H1N1)pdm09 season 2015‐2016 (120 573 (Table 1). For children less than 5 years old, the annual number of MCIC was highest in the A(H3N2) season 2011‐2012, with 74 517 MCIC.

#### 3.2.2 SHCIC, ICU admissions and influenza deaths in secondary care

**Figure 4:**
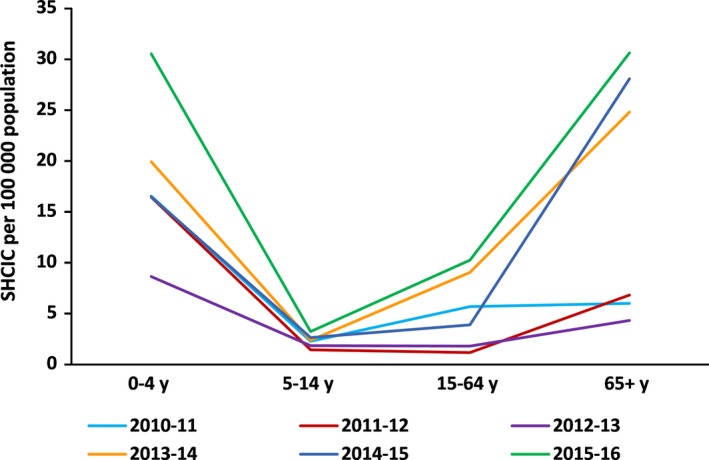
Secondary care. Cumulative annual rates of severe hospitalized confirmed influenza cases by age group, surveillance of SHCIC, Spain

Surveillance in secondary care evidenced for all the influenza seasons the highest annual cumulative SHCIC in the extreme age‐groups: 0‐4 and 65+ years old. We estimated between 8 and 30 hospitalizations per 100 000 population for the children up to 4 years old and between 4 and 30 hospitalizations per 100 000 population in the elderly group, depending on the influenza season (Figure 4).

In the study period, there was an annual average of 3616 SHCIC (Table 2). On average, the estimated severe hospitalized rates were highest in children under 4 years of age (18.9 SHCIC/100 000 population) and in elderly (16.5 SHCIC/100 000 population). ICU admission rates were also highest in these age‐groups, with 4.9 ICU admissions/100 000 population and 4.5 ICU admissions/100 000 population in children <4 years and in elderly, respectively. There was an annual average of 437 influenza hospitalized deaths. Annual influenza hospitalized rates were highest in the elderly (3 deaths in influenza hospitalized patients/100 000 population) (Table 2).

**Table 2:** Annual average number and cumulative rates of severe hospitalized confirmed influenza cases, ICU admissions, and deaths in influenza hospitalized patients, Spain, 2010‐2011 to 2015‐2016

Age‐group	SHCIC			ICU admissions			Deaths in influenza hospitalized patients		
	N	Rate per 100 000 pop	95% CI	N	Rate per 100 000 pop	95% CI	N	Rate per 100 000 pop	95% CI
All ages	3616	7.8	7.5‐8.0	1232	2.6	2.5‐2.8	437	0.9	0.8‐1.0
Aged 65+	1403	16.5	15.7‐17.4	383	4.5	4.1‐5.0	254	3.0	2.6‐3.4
15‐64 y	1651	5.3	5.1‐5.6	700	2.3	2.1‐2.4	170	0.5	0.4‐0.6
5‐14 y	107	2.3	1.9‐2.7	33	0.7	0.5‐1.0	3	0.1	0.0‐0.2
0‐4 y	426	18.9	17.1‐20.8	110	4.9	4.0‐5.9	4	0.2	0.1‐0.4

The number of SHCIC in Spain in the study period ranged between 1221 in the 2012‐2013 and 6694 in the 2015‐2016 season; the number of ICU admissions ranged between 464 in 2011‐2012 and 2312 in 2015‐2016 season; the number of deaths in influenza hospitalized patients ranged between 115 in 2011‐2012 and 760 in 2015‐2016 season (Table 3).

**Table 3:** Severe hospitalized confirmed influenza cases, ICU admissions and deaths in influenza hospitalized patients by age‐group, surveillance of SHCIC, Spain, seasons 2010‐2011 to 2015‐2016

Season	Dominant influenza type/subtype	SHCIC			ICU admissions			Deaths in influenza hospitalized patients		
		Number	95% CI		Number	95% CI		Number	95% CI	
All ages										
2010/11	A(H1N1)2009	2751	2649	2856	1017	955	1081	325	291	362
2011/12	A(H3N2)	1405	1332	1480	464	423	508	115	95	138
2012/13	B	1221	1153	1291	472	430	517	126	105	150
2013/14	A(H1N1)2009	5483	5339	5630	1874	1790	1961	667	617	720
2014/15	A(H3N2)	4139	4014	4267	1251	1183	1322	629	581	680
2015/16	A(H1N1)2009	6694	6535	6856	2312	2219	2408	760	707	816
Aged 65+										
2010/11	A(H1N1)2009	472	430	517	137	115	162	90	72	111
2011/12	A(H3N2)	546	501	594	189	163	218	73	57	92
2012/13	B	352	316	391	116	96	139	69	54	87
2013/14	A(H1N1)2009	2052	1964	2143	553	508	601	393	355	434
2014/15	A(H3N2)	2366	2272	2463	586	540	635	465	424	509
2015/16	A(H1N1)2009	2632	2532	2735	714	663	768	433	393	476
15‐64 y										
2010/11	A(H1N1)2009	1777	1695	1862	762	709	818	226	197	257
2011/12	A(H3N2)	371	334	411	159	135	186	35	24	49
2012/13	B	567	521	616	277	245	312	47	35	63
2013/14	A(H1N1)2009	2825	2722	2931	1169	1103	1238	258	227	291
2014/15	A(H3N2)	1205	1138	1275	506	463	552	143	121	168
2015/16	A(H1N1)2009	3160	3051	3272	1327	1257	1400	313	279	350
5‐14 y										
2010/11	A(H1N1)2009	102	83	124	26	17	38	2	0	7
2011/12	A(H3N2)	65	50	83	18	11	28	2	0	7
2012/13	B	85	68	105	24	15	36	2	0	7
2013/14	A(H1N1)2009	111	91	134	35	24	49	7	3	14
2014/15	A(H3N2)	126	105	150	41	29	56	5	2	12
2015/16	A(H1N1)2009	155	132	181	53	40	69	2	0	7
0‐4 y										
2010/11	A(H1N1)2009	404	366	445	95	77	116	5	2	12
2011/12	A(H3N2)	411	372	453	92	74	113	2	0	7
2012/13	B	210	183	240	54	41	70	5	2	12
2013/14	A(H1N1)2009	471	429	516	113	93	136	4	1	10
2014/15	A(H3N2)	379	342	419	104	85	126	5	2	12
2015/16	A(H1N1)2009	683	633	736	204	177	234	2	0	7

Regarding the burden of severe influenza disease by age‐group, in influenza seasons with predominant A(H1N1)pdm09 circulation, we estimated the highest number of SHCIC in young adults: 1777, 2825, and 3160, for the 2010‐2011, 2013‐2014, and 2015‐2016 influenza seasons, respectively (Table 3). ICU admissions and deaths in influenza hospitalized patients were also highest in those seasons. In children less than 5 years old, the season 2015‐2016 yielded the highest number of SHCIC and ICU admissions, 683 and 204, respectively. In elderly, we estimated the highest number of deaths in influenza hospitalized patients in the A(H3N2) 2014‐2015 season, accounting for 465 deaths (95%IC: 424‐509) (Table 3).

**Figure 5:**
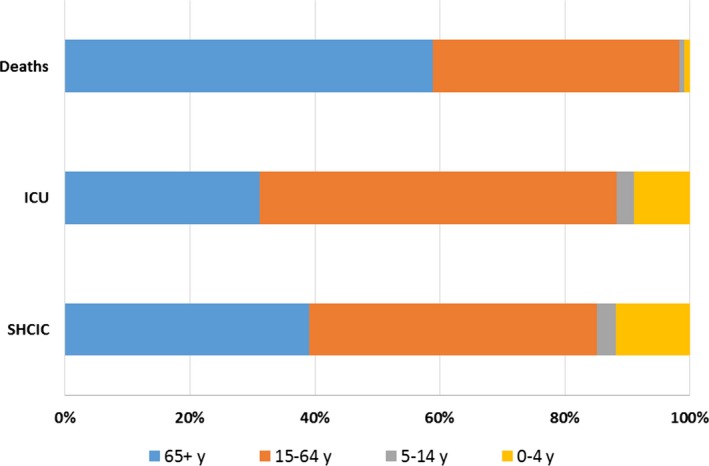
Percentage of severe hospitalized confirmed influenza cases, ICU admissions and deaths in influenza hospitalized patients by age group, Spain, seasons 2010–11 to 2015–16

Of the total estimated number of SHCIC and ICU admissions, 46% and 57%, respectively, were in the young adults group, followed by the elderly (39% and 31%). Only 23 deaths in hospitals were estimated for children less than 5 years old, while the majority of deaths occurred in the 65+ age‐group (58%), followed by the 15‐64 age‐group (39%) (Figure 5).

Within the 6 influenza seasons 2010‐2011 to 2015‐2016, the percentage of ICU admission among the SHCIC was highest (42%) in the 15‐64 age‐group, while the hospitalization fatality rate was highest in elderly (18% of deaths among SHCIC) (Table 4). Children 0‐4 and 5‐14 years old experienced a percentage of ICU admission of 31% and 26%, respectively, but the hospitalization fatality rate was very low (1%‐3%) compared to the young adults and elderly (10% and 18%, respectively) (Table 4).

**Table 4:** Number and percentage of ICU admissions and deaths in influenza hospitalized patients, Spain, 2010‐2011 to 2015‐2016

Age‐group	SHCIC	ICU admissions			Deaths in influenza hospitalized patients		
	N	N	Percentage of admission	95% CI	N	Hospitalization fatality rates	95% CI
All ages	21 693	7390	34.1	33.4‐34.7	2622	12.1	11.7‐12.5
Aged 65+	8420	2295	27.3	26.3‐28.2	1523	18.1	17.3‐18.9
15‐64 y	9905	4200	42.4	41.4‐43.4	1022	10.3	9.7‐10.9
5‐14 y	644	197	30.6	27.0‐34.3	20	3.1	1.9‐4.7
0‐4 y	2558	662	25.9	24.2‐27.6	23	0.9	0.5‐1.3

## 4 DISCUSSION

We estimated the burden of mild and severe seasonal influenza during the post‐2009 pandemic period in Spain, using information from influenza surveillance systems at the primary care and hospital level. The burden of seasonal influenza has been substantial in Spain, with an annual average of 866 868 ILI cases attended in sentinel primary care, of which 55% were influenza confirmed (474 607 MCIC). At the hospital level, we estimated an annual average of 3616 episodes of severe confirmed influenza hospitalizations, 1232 episodes of influenza ICU admissions, and 437 deaths in influenza hospitalized patients.

The SISSS and the surveillance of SHCIC are the only nationally systems for ILI patients attended in sentinel primary care and for severe influenza hospitalized patients, representative for the Spanish population, incorporating both epidemiological and virological surveillance. These systems have proved their usefulness to calculate the burden of influenza disease in Spain using routine surveillance data, allowing us to estimate the impact of mild and severe influenza infection in Spain after the 2009 influenza pandemic.

Our results indicate that the seasonal influenza burden of both mild and severe influenza disease in Spain is virus‐ and age‐specific, with significant seasonal variation. During the 6 studied influenza seasons, we found that, while children under the age of 15 years have the highest ILI and MCIC rates, the very young (0‐4 years) and the elderly exhibit the highest rates of severe influenza‐associated hospitalizations. This is consistent with previous studies showing that young children and adolescents have the highest rates of medically attended influenza,^7^, ^12^, ^13^, ^14^ while the highest hospitalization rates for influenza are observed among very young^15^ and persons aged ≥65 years.^16^ Previous exposure to influenza viruses, providing a partial immunity to the circulating viruses, and higher vaccine coverage than in other age‐groups,^17^, ^18^ could account for the lower ILI rates at the primary care level in the elderly population. In addition, as the elderly have also the highest prevalence of chronic conditions, they would be more prone to complications and hospitalizations, once acquiring the influenza infection, therefore yielding higher hospitalization rates in this population group.^19^

General primary care practitioners in Spain attended about half a million ILI cases each season in young adults (15‐64 years) and between 40 000 and 90 000 in persons aged ≥65 years old. Pediatricians attended each season about 100 000 ILI cases in children <5 years of age and 200 000 children aged 5‐14 years. We observed a higher burden of mild influenza for all ages groups in the 2014‐2015 season, dominated by the A(H3N2) influenza virus, when the annual cumulative influenza rate was estimated at 2367 ILI cases/100 000 population, the highest rate since the 2009 pandemic, suggesting a high transmissibility of the virus A(H3N2) during 2014‐2015 epidemic.^20^ In elderly individuals, our findings suggest a higher burden of mild cases in the 2011‐2012 and 2014‐2015 influenza seasons, dominated by A(H3N2), consistent with the high incidence of medically attended illness in this population previously reported by other authors in primary care during the same period.^21^ During the influenza seasons, where influenza B was predominant (2012‐2013 season) or presented at a late peak of activity (2014‐2015 and 2015‐2016 influenza seasons), we found that the 5‐14 age‐group was considerably more affected compared to other influenza seasons. This finding was also described in other countries in Europe and in Australia.^22^, ^23^, ^24^ In children younger than 5 years, it is more difficult to find a virus‐specific pattern regarding the influenza disease burden. We found a high disease burden in seasons dominated by A(H1N1) (2015‐2016), but also in A(H3N2) dominant as the 2011‐2012 influenza season. This larger burden in very young children was also reported in many countries in Europe during the 2011‐2012 season, in accordance with a limited immunity against A(H3N2) virus, especially in younger age‐groups, after several influenza seasons without circulation of this influenza virus.^25^

We have estimated the burden of severe influenza during each influenza epidemic finding an average of 3616 SHCIC, 1232 ICU admissions, and 437 deaths in severe influenza hospitalized patients. The impact of severe influenza varied by age‐group and across the 6 studied influenza seasons.

As we mentioned above, the impact of severe influenza during the 6 studied influenza seasons, as measured by the cumulative hospitalization rate, was higher in Spain for children less than 5 years old and elderly. However, in terms of the number of severe influenza cases, young adults bore the largest burden of influenza hospitalization and ICU admission, followed by the elderly. These 2 groups accounted for 85% and almost 90% of the total number of SHCIC and patients admitted to ICU, respectively. Mortality was highest in the elderly with 58% of the deaths reported in influenza hospitalized patients, followed by 39% in young adults. Future studies are needed to explore if certain risk factors contribute to mortality in the young adults group (e.g, underlying conditions).

Overall, during the post‐2009 influenza pandemic period, the proportion of severe influenza cases admitted to the UCI was highest in young adults 15‐64 years old, consistent with other studies in the same period.^26^ In addition, the number of patients in this age‐group admitted to the ICU was higher in A(H1N1)pdm09 dominant seasons compared with other subtype seasons.^26^, ^27^

Children under 5 years of age experienced the highest number of SHCIC and ICU admissions during the season 2015‐2016, concordant with the high burden of mild disease in this age‐group observed in primary care in the 2015‐2016 season.^28^ However, there was no increase in the number of deaths in influenza hospitalized patients belonging to this age‐group.

In our study, influenza‐associated mortality increased with age, with the elderly having the highest hospitalization fatality rate, similarly to regression results describing the acute respiratory illness deaths attributable to influenza.^15^, ^29^ Consistently, we found the highest number of deaths in influenza hospitalized patients occurred in the elderly group in almost all seasons except the first post‐pandemic 2010‐2011 season. This was the only season when the number of deaths in severe influenza hospitalized patients in young adults was higher than in elderly. Moreover, higher numbers of deaths were observed in the young adults population in each season with important A(H1N1)pdm09 circulation, compared to other seasons. This is in line with the previously documented higher proportions of deaths among patients younger than 65 during the decade following each previous pandemic.^30^

The highest number of deaths in severe influenza hospitalized patients in the elderly was found in the 2014‐2015 influenza season dominated by A(H3N2), although high numbers of deaths were also observed for this age‐group in the preceding and subsequent A(H1N1)pdm09 seasons. It is accepted that influenza A(H3N2) can cause a relatively higher impact than other influenza viruses, mainly regarding the mortality within older age‐groups.^29^, ^31^ Interestingly, this was not reproduced in our study during the 2011‐2012 influenza season, dominated by A(H3N2) circulation. Some contributing factors might be related with limitations of the severe influenza surveillance in Spain, as we describe next.

The variation of the impact of severe influenza by season, for all age‐groups, is greater than that observed for mild disease. One of the reasons can be related with seasonal differences in influenza severity and/or the shorter time since the implementation of the surveillance system for SHCIC, but some other factors might have also influenced the findings. After the post‐pandemic 2010‐2011 influenza season, the perception of influenza threat and awareness of the hospital clinics contributing to the severe influenza surveillance, after more than 2 years with exhaustive work load due to “pandemic 2009”, might contribute to under‐notification of SHCIC which influenced the reported severe influenza burden in the 2011‐2012 and 2012‐2013 seasons. However, shortly before the peak of the 2013‐2014 epidemic, a local alert on severe influenza generated a huge media movement in Spain that changed the dynamic of the surveillance and increased the clinical awareness and therefore the recruitment and report of severe influenza cases to the system.^32^ Although we did not attempt to correct surveillance data for under‐detection, as other studies have carried out,^33^ the surveillance indicators did evidence an improvement in the SHCIC information reporting, assuming the consolidation of the system from the 2013‐2014 influenza season onwards. Thus, under‐ascertainment of SHCIC within the system may have occurred specially during the 2 seasons following the post‐pandemic 2010‐2011 influenza season, while a higher and more homogenous estimate of burden of severe influenza is found in this study for the last 3 studied seasons.^28^, ^34^

Several other limitations must be taken into account when interpreting our study results. Our estimation of severe influenza burden in Spain is restricted to surveillance data which includes severity criteria in the SHCIC case definition. Therefore, our results are conservative when compared to those based on the reporting of influenza hospitalized cases independently of its clinical severity,^35^ yielding lower hospitalization rates. This also applies to the number of deaths reported in influenza hospitalized patients. Results of regression models, previously applied in our group, gave an estimate of all causes excess mortality rate attributable to influenza of 170 deaths/100 000 population in the >64 age‐group for the 2011‐2012 influenza season.^36^ In contrast, during the 2011‐2012 influenza season, we registered 73 deaths in influenza hospitalized patients older than 64 years, yielding a rate of 1 death/100 000 population. Studies on the excess mortality attributable to influenza for the post‐2009 influenza pandemic period are ongoing and might provide additional knowledge regarding the impact of influenza epidemics on the mortality of the population in Spain.

A possible additional limitation when estimating the burden of severe disease can be derived from collecting only hospitalized cases and not those who are admitted for less than 24 hours to the emergence room. Likewise, admission to the ICU might be affected by clinical management criteria in the case of very elderly people.

Considering together the mentioned limitations, we believe our results underestimate the real burden of severe influenza disease in Spain. Nevertheless, updated influenza burden information will be essential to guide public health policy and control measures.

An underestimate of the burden of mild influenza may also have occurred, as the ILI case definition only captures a proportion of all symptomatic cases^37^ and is less sensitive compared to acute respiratory infection (ARI) case definition. However, the sentinel sample positivity is quite high in Spain compared to other countries and besides other factors this could be related to the high performance of ILI's ascertainment of the sentinel physicians belonging to the SISSS.^38^

Finally, direct comparisons with equivalent studies are difficult due to differences not only in the estimation methods, but also in the different case definitions used, surveillance protocols, sentinel physicians training, primary, and secondary healthcare systems characteristics, etc.

In conclusion, we report a considerable influenza disease burden in Spain, with significant seasonal and age variations. Children less than 15 years old yielded the highest influenza activity in primary care, while influenza hospitalization rates were highest among the very young children and the elderly. Nevertheless, the impact of influenza in the National Health System was mainly concentrated in young adults for mild influenza, and in young adults and elderly for severe influenza disease.

Estimates of mild and severe influenza disease burden in Spain will allow understanding the impact of the epidemics, including potential influenza pandemics, helping to guide decisions targeting influenza prevention and control. This information, periodically updated, will also be crucial to estimate the impact of influenza vaccination programs, contributing toward enhanced guidance for the development of long lasting and more efficient influenza vaccines.

## CONFLICT OF INTEREST

The authors report no competing interests.

## APPENDIX 1

### 1.1

The Spanish Influenza Surveillance System includes the following:

Physicians of the influenza sentinel surveillance networks of Andalucía, Aragón, Asturias, Baleares, Canarias, Cantabria, Castilla la Mancha, Castilla y León, Cataluña, Comunidad Valenciana, Extremadura, Madrid, Navarra, País Vasco, La Rioja, Ceuta and Melilla; Andalucía: Virtudes Gallardo (Servicio de Epidemiología, Consejería de Salud); Jose María Navarro (Hospital Virgen de las Nieves de Granada). Aragón: Elisa Marco (Servicio de Vigilancia en Salud Pública, Dirección General de Salud Pública). Manuel Omeñaca (Hospital Universitario Miguel Servet). Asturias: Ismael Huertas (Dirección General de Salud Pública y Planificación, Consejería de Salud y Servicios Sanitarios); María de Oña (Hospital Universitario Central de Asturias de Oviedo). Baleares: Jaume Giménez (Servicio de Epidemiología, Dirección General de Salut Pública); Jordi Reina (Hospital Son Espases de Palma de Mallorca). Canarias: Lucas González (Servicio de Epidemiología y Prevención, Consejería de Sanidad); Carmen Pérez (Hospital Dr Negrín de Las Palmas de Gran Canarias). Cantabria: Luis Viloria (Sección de Epidemiología, Consejería de Sanidad, Trabajo y Servicios Sociales); Mónica Gozalo (Hospital Universitario Marqués de Valdecilla). Castilla la Mancha: Gonzalo Gutiérrez (Servicio de Epidemiología, Consejería de Sanidad). Castilla y León: Tomás Vega (Observatorio de Salud Pública, DGSP, Consejería de Sanidad); Socorro Fernández (Servicio de Epidemiología, DGSP, Consejería de Sanidad); Raúl Ortiz de Lejarazu (National Influenza Centre, Hospital Clínico Universitario de Valladolid). Cataluña: Nuria Torner (Subdirecció General de Vigilància i Resposta a Emergències en Salut Pública, Agència de Salut Pública, CIBERESP); María de los Ángeles Marcos (National Influenza Centre, Hospital Clínic de Barcelona). Comunitat Valenciana: Aurora López (Subdirección General de Epidemiologia y Vigilancia de la Salud, Conselleria de Sanitat); Francisco González (Subdirección General de Epidemiologia y Vigilancia de la Salud, y Sanidad Ambiental. DGSP, Conselleria de Sanitat Universal i Salut Publica); Concepción Gimeno (Consorci Hospital General Universitari de València). Extremadura: Julián Mauro Ramos (Subdirección de Epidemiología. Dirección General de Salud Pública, Servicio Extremeño de Salud); Guadalupe Rodríguez (Complejo Hospitalario San Pedro de Alcántara, Cáceres). Galicia: María Jesús Purriños (Dirección Xeral Saúde Pública de Galicia); Juan García Costa (Complejo Hospitalario Universitario de Orense); Sonia Pérez‐Castro (Complejo Hospitalario Universitario de Vigo). Madrid: Luis García (Servicio de Epidemiología. Dirección General de Salud Pública); Juan Carlos Galán (Hospital Universitario Ramón y Cajal). Murcia: Rocío García (Servicio de Epidemiología, Consejería de Sanidad); Antonio Moreno (Hospital Virgen de la Arrixaca de Murcia). Navarra: Jesús Castilla (Sección de Vigilancia de Enfermedades Transmisibles del Instituto de Salud Pública, CIBERESP); Mirian Fernández‐Alonso (Clínica Universidad de Navarra); Carmen Ezpeleta (Complejo Hospitalario de Navarra). País Vasco: Fernando González Carril (Servicio de Salud Pública, Departamento de Salud); Gustavo Cilla (Hospital Donostia de San Sebastián, CIBERES). La Rioja: Carmen Quiñones (Servicio de Epidemiología y Prevención Sanitaria, Dirección General de Salud Pública y Consumo); Miriam Blasco (Hospital San Pedro de Logroño). Ceuta: Ana Rivas (Sección de Vigilancia Epidemiológica, Consejería de Sanidad y Bienestar Social); José López (Hospital Universitario de INGESA). Melilla: Daniel Castrillejo (Servicio de Epidemiología, Dirección General de Sanidad y Consumo, Consejería de Bienestar Social y Sanidad). Francisco Pozo, Inmaculada Casas, (National Centre for Microbiology, National Influenza Reference Laboratory, National Influenza Centre, ISCIII). Salvador de Mateo (National Centre of Epidemiology, CIBERESP, ISCIII).
